# Frequency of β-Casein Gene Polymorphisms in Jersey Cows in Western Japan

**DOI:** 10.3390/ani12162076

**Published:** 2022-08-15

**Authors:** Nuomin, Qui Dang Nguyen, Aodaohu, Naoki Nishino

**Affiliations:** Department of Animal Science, Graduate School of Environmental and Life Science, Okayama 700-8530, Japan

**Keywords:** A2 milk, β-casein, Jersey, plasma metabolites, polymorphism

## Abstract

**Simple Summary:**

Reports presenting survey results for β-casein gene polymorphisms have increased, but none have been about Jersey cows in Asia. This study examined the *CSN2* gene variants for 590 Jersey cows in Okayama Prefecture, located in the western region of Japan. Blood samples were collected at eight farms, and nucleotide substitutions were determined by sequencing exon 7 regions of chromosome 6 of the *CSN2* gene. Blood biochemical analyses were also performed to clarify if A1A1, A1A2, and A2A2 cows differ in their metabolic profiles. The frequency of the A2 allele found in this study was numerically higher than those reported for Holsteins, crossbreeds, and Mexican and Danish Jerseys. The β-casein genotypes did not affect the metabolism of the major nutrients.

**Abstract:**

This study aimed to investigate β-casein gene polymorphisms in Jersey cows in Japan. Blood samples were collected from 590 cows from eight Jersey farms in Okayama Prefecture, western Japan. Sequence analysis of exon 7 regions in chromosome 6 of the *CSN2* gene revealed the genotype and allele frequencies of the β-casein variants. Considering that variant B belongs to the A1 group and variant I to the A2 group, plasma metabolite concentrations were compared among the A1A1, A1A2, and A2A2 group-based genotypes. The most frequent genotype was A2A2 (0.558), followed by A2B (0.190) and A2I (0.103). No variants of A3, F, G, H1, or H2 were found. The frequencies of group-based genotypes were A1A1 (0.032), A1A2 (0.303), and A2A2 (0.665). Although farm-to-farm differences were observed in the plasma concentrations of urea nitrogen, calcium, and phosphorus, no differences were found between the A1A1, A1A2, and A2A2 group-based genotypes; hence, the β-casein genotypes did not affect the metabolism of major nutrients. Owing to the high frequency of the A2 variant, Jersey cows can be considered an attractive breed for marker-assisted selection to create A2A2 herds.

## 1. Introduction

Bovine milk contains many essential nutrients and is a common source of protein for humans. Approximately 80% of the milk protein is casein, of which four polymorphisms, αs1-, αs2-, β-, and κ-casein, encoded by *CSN1S1*, *CSN1S2*, *CSN2*, and *CSN3* genes, respectively, are described [1,2]. As1-casein is the most abundant (~38% of total caseins), followed by β-casein (~36%), κ-casein (~13%), and αs2-casein (~10%) [2]. Β-casein consists of 209 amino acid residues that can be divided into 12 variants based on genetic polymorphism [3]: A1, A2, A3, B, C, D, E, F, G, H1, H2, and I (Table 1). The A2 variant is considered the oldest and the original variant and has a proline at position 67 of the β-casein chain [4]. In the A1 variant, proline is substituted by histidine. The most common variants for Holsteins were A1 and A2, and the B and I variants were usually detected at lower frequencies [1]. The A3, D, E, H1, H2, and I variants can be referred to as the A2 group, as they also have a proline at position 67 [3,4]. Likewise, the B, C, F, and G variants that have histidine at position 67 can be regarded as the A1 group. Only two alleles (A1 and A2) have been shown in several reports, in which substitutions of nucleotides specific to other variants were not examined.

β-casein is characterized by a large number (35 out of 209 amino acids in the A2 variant) of proline, a cyclic amino acid that complicates the formation of proteins’ secondary structure. Because of this mutation, β-casein variants A1 and A2 may be cleaved differently during digestion and food processing. Digestive enzymes cannot perform proteolytic cleavage of the β-casein chain at position 67 in the presence of proline [1]. However, the bond can be cleaved when proline is substituted with histidine, releasing a peptide of seven amino acids (Tyr-Pro-Phe-Pro-Gly-Pro-Ile) called β-casomorphin 7 (BCM-7). BCM-7 is a bioactive peptide with morphine-like activity that has been linked to several diseases, such as type 1 diabetes and ischemic heart disease [1,5,6]. Furthermore, adverse gastrointestinal effects from milk consumption, such as diarrhea, bloating, and abdominal pain, which have been related to insufficient lactose digestion, have also been considered to be related to β-casein A1 variant consumption. Although scientific evidence supporting these health effects is lacking and is under debate [5,6], the demand for milk produced from A2A2 genotype cows (A2 milk) has increased.

Many reports have presented survey results for β-casein gene polymorphisms. Differences due to breed and region are apparent because the frequency of the A1 variant could be the consequence of improved breeding. Yamada et al. [7] identified A1, A2, and B variants in 390 cows in Japan, most of which were Holsteins. Because more than 99% of the dairy cow population in Japan comprises Holsteins, the survey reflected the current β-casein polymorphism in Japanese dairy cows. Jerseys make up <1% of cows in Japan; however, they produce milk that is rich in protein and fat and are thus are favored as a breed suitable for manufacturing distinctive milk and milk products. Okayama Prefecture, located in the western region of Japan, is a central area for Jersey farming. Crossbreeding of Holsteins and Jerseys is rarely observed in Japan; hence, almost all Holsteins and Jerseys are pure breeds.

This study aimed to investigate β-casein gene polymorphisms in Jersey cows in Okayama Prefecture, Japan. A total of 590 cows from eight farms were examined. Nucleotide substitutions were determined by sequencing exon 7 regions of chromosome 6 of the *CSN2* gene. Four variants (A1, A2, B, and I) were detected, and polymorphisms were described in their genotypes and group-based (A1A1, A1A2, and A2A2) genotypes. Furthermore, blood biochemical analyses were performed on the cows in two farms to clarify if A1A1, A1A2, and A2A2 cows differ in their metabolic profiles.

## 2. Materials and Methods

Blood samples of Jersey cows were collected from eight farms in Okayama Prefecture, in western Japan, during October and November 2019 and 2020 (Table 2). Sampling was performed in separate tubes containing heparin as an anticoagulant at the same time that the periodic diagnostic tests for Johne’s disease were performed. The tubes were kept on ice until processing in the laboratory. All procedures and protocols for animal experiments were approved by the Animal Care and Use Committee, Okayama University (OKU-2020856), Japan.

After centrifugation at 650× *g* for 10 min, the plasma and buffy coat were collected and stored at −30 °C. Genomic DNA was extracted using the DNeasy Blood and Tissue Kit (Qiagen, Tokyo, Japan). The exon 7 regions of the *CSN2* gene were amplified using forward (5′-TTTCCAGGATGAACTCCAGGAT-3′) and reverse (5′-CATCAGAAGTTAAACAGGCACAGTTAG-3′) primers [3]. PCR was conducted using a SureDirect Blood PCR kit (Agilent, Tokyo, Japan), and sequencing was carried out using a BrilliantDye^TM^ Terminator (v3.1) Cycle Sequencing Kit (NimaGen, Nijmegen, The Netherlands). DNA base sequences were analyzed using an ABI PRISM^®^ 3130xl Genetic Analyzer (Thermo Fisher Scientific Inc., Tokyo, Japan). Electropherograms were examined at the mutation points to discriminate between homozygous and heterozygous peaks. Polymorphisms at positions 67, 72, 88, 93, 106, 122, and 138 of exon 7 were analyzed to detect β-casein variants. 

The concentrations of plasma albumin, urea nitrogen (BUN), total cholesterol, non-esterified fatty acids (NEFA), aspartate aminotransferase (AST), alanine aminotransferase (ALT), calcium (Ca), and phosphorus (P) were determined using their respective commercial kits (FUJIFILM Wako Pure Chemicals Co., Tokyo, Japan). The protein, fat, solids-not-fat, urea nitrogen (MUN) content, and somatic cell count (SCC) of bulk milk were determined using a CombiFoss FT+ analyzer (Foss Allé, Hillerød, Denmark). The composition of bulk milk was monitored three times per month on the eight farms that were surveyed.

Genotype and allele frequencies were calculated by dividing the number of copies of each genotype and allele by the total cows examined and the total alleles detected, respectively. The Hardy–Weinberg equilibrium was verified using the chi-square test. Data for the concentration of blood metabolites were analyzed by two-way analysis of variance with genotype and farm as main factors. 

## 3. Results and Discussion

Sequence analysis of exon 7 regions of the *CSN2* gene detected four β-casein variants (A1, A2, B, and I) in 12 variants at all eight farms (Table 3). Variants A3, F, G, H1, and H2 were not found in Jersey herds in the present study. The frequencies of homozygous (A1A1, BB, A2A2, and II) and heterozygous (A1A2, A1B, A1I, A2B, A2I, and BI) alleles were different between farms. One farm (F5) had all ten genotypes; three farms (F3, 4, and 8) lacked A1A1, A1B, BB, and A2I genotypes; and six farms (F1, 2, 3, 4, 6, and 7) lacked the II genotype. 

The A2 allele was the most frequently found across the eight farms, but the second most frequent allele was B or I, depending on the farm. The average frequencies of A1, A2, B, and I alleles were 0.059, 0.746, 0.125, and 0.070, respectively. Although no A3 variant was found in this study, the A3 allele was observed at frequencies of 0.005, 0.004, and 0.001 for Danish Holstein [8], Chinese Holstein [9], and Italian Holstein [3] cows, respectively. Likewise, although the average frequency of the B allele was 0.125 in this study, the allele was usually detected at 0.04–0.06 for Holsteins [3,7,9]. Reports of β-casein variants in Jersey cows are limited. Zepeda-Baldomero et al. [10] found the A1, A2, A3, and B allele frequencies to be 0.219, 0.691, 0.040, and 0.040, respectively, in Mexican Jerseys. Likewise, Gustavsson et al. [8] detected the A1, A2, B, and I alleles at 0.081, 0.634, 0.218, and 0.067, respectively, in Danish Jerseys. The findings that the A3 variant was not found and the B variant appeared more frequently than Holsteins in Danish Jerseys were similar to our results.

Our data were compared with other published data based on group-based genotypes, considering that the B, C, F, and G variants are the A1 group and the A3, D, E, H1, H2, and I variants are the A2 group (Table 4). The frequency of the A2 allele (0.816) in this study was numerically higher than those reported for Holsteins (0.508–0.744) [3,7,8,9,11,12,13], crossbreeds (0.568–0.606) [14,15,16], Mexican Jerseys (0.738) [10], and Danish Jerseys (0.700) [8]. If the herd aims to produce milk only from the A2 variant (A2 milk), a high frequency of the A2 allele is favored for selected breeding. In this regard, except for the extremely high frequencies of the Gir (0.956) and Guzera (0.932) breeds reared in Brazil [17], Jerseys in Japan can be advantageous over Holsteins and Jerseys in other regions. Interestingly, the frequencies of the A2 allele were numerically low for Polish Reds (0.370) [18] and Swedish Reds (0.508) [8].

Evidence suggests a relationship between β-casein variants, milk productivity, and milk composition. Olenski et al. [19] reported that the A2 allele is positively related to milk yield and milk protein yield (not milk protein content), and that the A1 allele is positively related to milk fat content (not milk fat yield). Visker et al. [20] indicated that the I allele is positively correlated with milk yield and milk protein content. Ivankovic et al. [12] found that the effect of β-casein variants was different in primiparous and multiparous cows; higher milk fat content for A1A1 cows was observed in the first lactation, and greater milk yield for A2A2 cows was observed in the second lactation. In this study, only eight farms were surveyed, and the milk yield and composition were recorded on a farm basis. Nevertheless, when the relationship between allele frequencies and milk productivity was analyzed (n = 8), the A1 allele was found to be positively correlated with milk fat content (r = 0.758, *p* < 0.05), and the I allele was negatively correlated with milk fat (r = −0.641, *p* < 0.05) and lactose (r = −0.653, *p* < 0.05) contents. Although our finding could be considered informative because of the insufficient number of observations, the positive relationship between the A1 allele and milk fat content is consistent with the findings of Olenski et al. [19].

**Figure 1:**
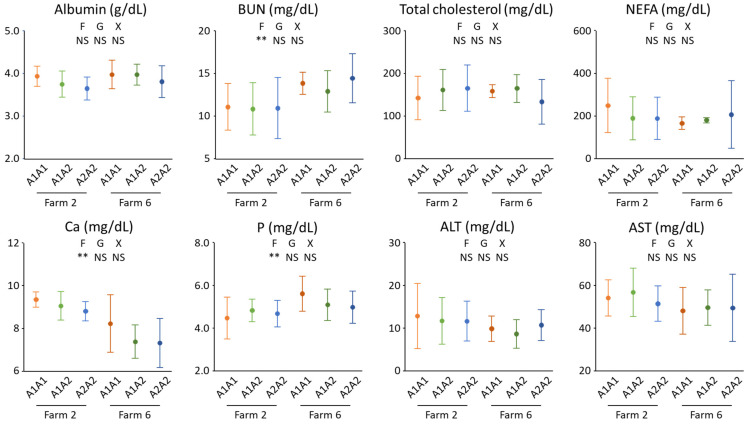
Plasma concentrations of albumin, urea nitrogen (BUN), total cholesterol, non-esterified fatty acid (NEFA), calcium (Ca), phosphorus (P), aspartate aminotransferase (AST), and alanine aminotransferase (ALT) of the Jersey cows from two farms in Okayama Prefecture, a western region of Japan. On each farm, blood samples were collected from six, ten, and ten cows of the A1A1, A1A2, and A2A2 β-casein variants, respectively. A two-way analysis of variance was performed to examine the effect of farm (F), genotype (G), and their interaction (X). NS; not significant (*p* ≥ 0.05), *; *p* < 0.05, **; *p* < 0.01.

The A1 allele was rarely observed in Jersey herds examined in this study. Even if A1A1, A1A2, and A2A2 group-based genotypes were applied, there were no A1A1 cows on the three farms (F3, 4, and 8). The maximum number of A1A1 cows on one farm was only six (F2 and 6); hence, blood biochemical analyses were restricted to Jersey cows on the two farms (Figure 1). Samples of three, three, and one A1A1 cow from other farms (F1, 5, and 7) were not examined because farm-to-farm differences may obscure differences due to genotypes in blood metabolite concentrations. Indeed, BUN and P concentrations were higher for the cows on one farm (F6), and the Ca concentration was greater for the cows on another farm (F2). Milk protein and MUN contents were numerically higher for bulk milk at F6 than at F2; hence, a higher BUN level implied more protein supply for the herd on F6. Meanwhile, no differences were observed in albumin, BUN, cholesterol, NEFA, AST, ALT, Ca, and P concentrations; hence, the β-casein genotypes did not affect the metabolism of the major nutrients. 

## 4. Conclusions

Awareness and demand for A2 milk have not been high in Japan until now, but a limited number of small-scale producers have pioneered its production. To date, all A2 milk is manufactured from Holstein milk. Considering the high frequency of the A2 variant in Jerseys compared with Holsteins, selective breeding to create A2A2 herds could be considered an attractive investment to increase the value of milk and milk products.

## Figures and Tables

**Table 1:** Changes in the amino acid sequence in the bovine β-casein variants.

Variant	Exon 6					Exon 7						
	18	25	35	36	37	67	72	88	93	106	122	138
A1						His						
A2	Ser-P	Arg	Ser-P	Glu	Glu	Pro	Gln	Leu	Met	His	Ser	Pro
A3										Gln		
B						His					Arg	
C			Ser		Lys	His						
D	Lys											
E				Lys								
F						His						Leu
G						His					Leu	
H1		Cys						Ileu				Glu
H2							Glu		Leu			
I									Leu			

The A1, A2, A3, B, F, G, H1, H2, and I variants were determined by nucleotide mutations in exon 7 regions of the *CSN2* gene in this study.

**Table 2:** Milk yield and composition of Jersey herds from eight farms examined in this study.

	Date of Blood Sampling	Yield (kg/day/cow)	Protein (%)	Fat (%)	SNF (%)	Lactose (%)	SCC (X10^3^ cells/mL)	MUN (mg/mL)
F1 (115)	29 October 2019	21.6 ± 0.42	4.07 ± 0.12	5.06 ± 0.23	9.54 ± 0.07	4.55 ± 0.05	205 ± 30.5	8.14 ± 0.88
F2 (82)	24 November 2020	23.6 ± 0.72	3.98 ± 0.18	5.20 ± 0.20	9.42 ± 0.16	4.51 ± 0.02	115 ± 22.0	7.59 ± 1.04
F3 (39)	20 October 2020	16.0 ± 0.51	4.17 ± 0.20	4.96 ± 0.29	9.59 ± 0.21	4.45 ± 0.02	351 ± 104	11.9 ± 1.31
F4 (98)	25 November 2020	23.7 ± 0.65	4.02 ± 0.13	5.01 ± 0.20	9.49 ± 0.14	4.55 ± 0.03	148 ± 62.6	8.90 ± 1.12
F5 (43)	19 October 2020	18.9 ± 0.67	4.10 ± 0.09	4.95 ± 0.25	9.48 ± 0.07	4.45 ± 0.01	265 ± 22.0	11.1 ± 0.47
F6 (60)	24 November 2020	21.7 ± 0.98	4.19 ± 0.11	5.27 ± 0.20	9.68 ± 0.12	4.54 ± 0.03	172 ± 22.9	9.60 ± 0.83
F7 (94)	11 November 2020	24.4 ± 0.75	3.96 ± 0.17	4.98 ± 0.11	9.40 ± 0.15	4.51 ± 0.03	207 ± 17.6	9.86 ± 1.34
F8 (59)	25 November 2020	18.8 ± 1.35	4.03 ± 0.14	4.66 ± 0.23	9.38 ± 0.17	4.43 ± 0.06	327 ± 43.6	10.9 ± 2.61

Mean values with standard deviations for four-month milking records (September, October, November, and December) are indicated. F1 stands for farm 1. The numbers in parentheses indicate the number of cows examined. Blood metabolite analyses were performed for samples from F2 and F6. SNF; solid-not-fat, SCC; somatic cell count, MUN; milk urea nitrogen.

**Table 3:** Genotype and allele frequencies of β-casein genes in Jersey herds in Okayama Prefecture, western Japan.

	Genotype Frequency										Allele Frequency			
	A1A1	A1B	BB	A1A2	A1I	A2B	BI	A2A2	A2I	II	A1	A2	B	I
F1 (115)	0.009	0.017	0	0.069	0.009	0.243	0.061	0.496	0.096	0	0.057	0.700	0.161	0.082
F2 (82)	0	0.061	0.012	0.098	0	0.244	0.000	0.512	0.073	0	0.079	0.719	0.165	0.037
F3 (39)	0	0	0	0.077	0	0.102	0.026	0.641	0.154	0	0.038	0.808	0.064	0.090
F4 (98)	0	0	0	0.092	0	0.092	0.020	0.704	0.092	0	0.046	0.842	0.056	0.056
F5 (43)	0.023	0.023	0.023	0.070	0.023	0.140	0.023	0.442	0.210	0.023	0.082	0.651	0.116	0.151
F6 (60)	0.017	0.067	0.017	0.116	0	0.266	0.017	0.483	0.017	0	0.108	0.683	0.192	0.017
F7 (94)	0	0.011	0	0.074	0	0.160	0.021	0.606	0.128	0	0.043	0.787	0.096	0.074
F8 (59)	0	0	0	0.068	0	0.237	0.034	0.525	0.119	0.017	0.034	0.737	0.136	0.093
Total (590)	0.005	0.022	0.005	0.083	0.003	0.190	0.027	0.558	0.104	0.003	0.059	0.746	0.125	0.070

The A3, F, G, H1, and H2 variants were not detected. F1 stands for farm 1. The numbers in parentheses indicate the number of cows examined.

**Table 4:** Comparison between reported studies of genotype and allele frequencies of β-casein genes in Holstein, Jersey, and other breeds.

References	Year	Country	Breed	Genotype Frequency			Allele Frequency	
				A1A1	A1A2	A2A2	A1	A2
This study	2022	Japan	Jersey (590)	0.032	0.304	0.664	0.184	0.816
Antonopoulos et al.	2021	Greece	Holstein (780)	0.033	0.445	0.522	0.256	0.744
			Vrachykeratiki (46)	0.000	0.609	0.391	0.304	0.696
Ivankovic et al.	2021	Croatia	Simmental (60)	0.033	0.417	0.550	0.242	0.758
			Holstein (60)	0.134	0.433	0.433	0.350	0.650
			Brown Swiss (60)	0.067	0.517	0.416	0.325	0.675
Mohan et al.	2021	India	Karan Fries (Holstein Friesian × Tharparkar crossbreed) (100)	0.090	0.620	0.290	0.400	0.600
Rangel et al.	2017	Brazil	Gir (68)	0.000	0.044	0.956	0.022	0.978
			Guzera (88)	0.000	0.068	0.932	0.034	0.966
Yamada et al.	2021	Japan	Holstein (311) & other three breeds (9)	0.141	0.441	0.418	0.361	0.639
Sebastiani et al.	2020	Italy (central)	Holstein Friesian (1629)	0.132	0.458	0.410	0.361	0.639
Kumar et al.	2020	India	Frieswal (Friesian × Sahiwal crossbreed) (429)	0.175	0.515	0.310	0.432	0.568
Cieslinska et al.	2019	Poland	Polish Red (177)	0.367	0.526	0.107	0.630	0.370
Massella et al.	2017	Italy (northern)	Holstein Friesian (1226) & Braunvieh (4)	0.185	0.487	0.328	0.428	0.572
Dai et al.	2016	China	Holstein (133)	0.271	0.444	0.285	0.492	0.508
Zepeda-Batista et al.	2015	Mexico	Jersey (453)	0.080	0.364	0.556	0.262	0.738
Gustavsson et al.	2014	Sweden	Holstein (415)	0.089	0.446	0.465	0.312	0.688
			Swedish Red (392)	0.230	0.525	0.245	0.492	0.508
			Jersey (406)	0.108	0.382	0.510	0.299	0.701
Molee et al.	2011	Thailand	Holstein × *Bos indicus* crossbreed (231)	0.074	0.640	0.286	0.394	0.606

When variants, in addition to A1 and A2, were reported, the frequencies were recalculated after group-based genotype classification. The numbers in parentheses are the number of cows examined.

## Data Availability

Raw data are stored in private computers and are available upon request.

## References

[1] Kaminski S., Cieslinska A., Kostyra E. (2007). Polymorphism of bovine beta-casein and its potential effect on human health. J. Appl. Genet..

[2] Elferink A.J.W., Entiriwaa D., Bulgarelli P., Smits N.G.E., Peters J. (2022). Development of a microsphere-based immunoassay authenticating A2 milk and species purity in the milk production chain. Molecules.

[3] Sebastiani C., Argangeli C., Ciullo M., Torricelli M., Cinti G., Fisichella S., Biagetti M. (2020). Frequencies evaluation of β-casein gene polymorphisms in dairy cows reared in central Italy. Animals.

[4] Caroli A.M., Savino S., Bulgari O., Monti E. (2016). Detecting β-casein gene variation in bovine milk. Molecules.

[5] de Gaudry D.K., Lohner S., Schmucker C., Kapp P., Motschall E., Horrlein S., Roger C., Meerpohl J.J. (2019). Milk A1 β-casein and health-related outcomes in humans: A systematic review. Nutr. Rev..

[6] de Gaudry D.K., Lohner S., Bischoff K., Schmucker C., Hoerrlein S., Roeger C., Schwingshackl L., Meerpohl J.J. (2022). A1 and A2 beta-casein on health-related outcomes: A scoping review of animal studies. Eur. J. Nutr..

[7] Yamada A., Sugimura M., Kuramoto T. (2021). Genetic polymorphism of bovine beta-casein gene in Japanese dairy farm herds. Anim. Sci. J..

[8] Gustavsson F., Buitenhuis A.J., Johansson M., Bertelsen H.P., Glantz M., Poulsen A., Lindmark Mansson H., Stalhammar H., Larsen L.B., Bendixen C. (2014). Effects of breed and casein genetic variants on protein profile in milk from Swedish Red, Danish Holstein, and Danish Jersey cows. J. Dairy Sci..

[9] Dai R., Fang Y., Zhao W., Liu S., Ding J., Xu K., Yang L., He C., Ding F., Meng H. (2016). Identification of alleles and genotypes of beta-casein with DNA sequencing analysis in Chinese-Holstein cow. J. Dairy Res..

[10] Zepeda-Batista J.L., Alarcon-Zuniga B., Ruiz-Flores A., Nunez-Dominguez R., Ramirez-Valverde R. (2015). Polymorphism of three milk protein genes in Mexican Jersey cattle. Electron. J. Biotechnol..

[11] Antonopoulos D., Vougiouklaki D., Laliotis G.P., Tsironi T., Valasi I., Chatzilazarou A., Halvatsiotis P., Houhoula D. (2021). Identification of polymorphisms of the CSN2 gene encoding beta-casein in Greek local breeds of cattle. Vet. Sci..

[12] Ivankovic A., Pecina M., Ramljak J., Pasic V. (2021). Genetic polymorphism and effect on milk production of CSN2 gene in conventional and local cattle breeds in Croatia. J. Dairy Prod. Process Improv..

[13] Massella E., Piva S., Giacometti F., Liuzzo G., Zambrini A.V., Serraino A. (2017). Evaluation of bovine beta casein polymorphism in two dairy farms located in northern Italy. Ital. J. Food Saf..

[14] Mohan G., Kumar A., Khan S.H., Kumar N.A., Kapila S., Lathwal S.S., Sodhi M., Niranjan S.K. (2021). Casein (CSN) gene variants and parity affect the milk protein traits in crossbred (Bos Taurus X Bos indicus) cows in sub-tropical climate. Trop. Anim. Health Prod..

[15] Kumar A., Singh R.V., Chauhan A., Ilayakumar K., Kumar S., Kumar A., Sonwane A., Kumar S., Panigrahi M., Bhushan B. (2020). Genetic association analysis reveals significant effect of β-casein A1/A2 loci on production and reproduction traits in Frieswal crossbred cows. Biol. Rhythm Res..

[16] Molee A., Boonek L., Rungsakinnin N. (2011). The effect of beta and kappa casein genes on milk yield and milk composition in different percentages of Holstein in crossbred dairy cattle. Anim. Sci. J..

[17] Rangel A.H.N., Zaros L.G., Lima T.C., Borba L.H.F., Novaes L.P., Mota L.F.M., Silva M.S. (2017). Polymorphism in the beta casein gene and analysis of milk characteristics in Gir and Guzera dairy cattle. Genet. Mol. Res..

[18] Cieslinska A., Fiedorowicz E., Zwiezchowski G., Kordulewska N., Jarmolowska B., Kostyra E. (2019). Genetic polymorphism of β-casein gene in Polish Red cattle-Preliminary study of A1 and A2 frequency in genetic conservation herd. Animals.

[19] Olenski K., Kaminski S., Szyda J., Cieslinska A. (2010). Polymorphism of the beta-casein gene and its associations with breeding value for production traits of Holstein-Friesian bulls. Live Sci..

[20] Visker M.H.P.W., Dibbits B.W., Kinders S.M., van Valenberg H.J.F., van Arendonk J.A.M., Bovenhuis H. (2010). Association of bovine β-casein protein variant I with milk production and milk protein composition. Anim. Genet..

